# New boronate drugs and evolving NDM-mediated beta-lactam resistance

**DOI:** 10.1128/aac.00579-23

**Published:** 2023-08-31

**Authors:** Olga Lomovskaya, Ruslan Tsivkovski, Maxim Totrov, Dana Dressel, Mariana Castanheira, Michael Dudley

**Affiliations:** 1 Qpex Biopharma, Inc., San Diego, California, USA; 2 Molsoft, LLC, San Diego, California, USA; 3 IHMA, Inc., Schaumburg, Illinois, USA; 4 JMI Laboratories, North Liberty, Iowa, USA; University of Fribourg, Fribourg, Switzerland

**Keywords:** metallo-β-lactamase, NDM, NDM-9, xeruborbactam, taniborbactam

## Abstract

Taniborbactam and xeruborbactam are dual serine-/metallo-beta-lactamase inhibitors (BLIs) based on a cyclic boronic acid pharmacophore that undergo clinical development. Recent report demonstrated that New Delhi metallo-beta-lactamase (NDM)-9 (differs from NDM-1 by a single amino acid substitution, E152K, evolved to overcome Zn (II) deprivation) is resistant to inhibition by taniborbactam constituting pre-existing taniborbactam resistance mechanism. Using microbiological and biochemical experiments, we show that xeruborbactam is capable of inhibiting NDM-9 and propose the structural basis for differences between two BLIs.

## INTRODUCTION

Recent studies suggest that NDM [metallo-beta-lactamase (MBL) of the New Delhi group] variants are evolving to overcome Zn (II) deprivation, a condition that can be elicited by the immune system in response to infection (1). One of these variants is NDM-9 that differs from NDM-1 by a single amino acid substitution, E152K (2), and has been reported in several species of *Enterobacterales* worldwide (3). Recent report (4) demonstrated that NDM-9 is resistant to inhibition by taniborbactam, a dual serine- (SBL)/metallo-beta-lactamase inhibitor (BLI) based on a cyclic boronic acid pharmacophore (5) that has completed Phase 3 clinical development in combination with cefepime (NCT03840148). With their study, the authors raised awareness of differences in sensitivity of MBLs to new inhibitors, including pre-existing resistance to cefepime-taniborbactam. This finding is of concern as it demonstrates that NDM-9 has an advantage either in the presence or in the absence of selective pressure from cefepime-taniborbactam.

**Fig 1:**
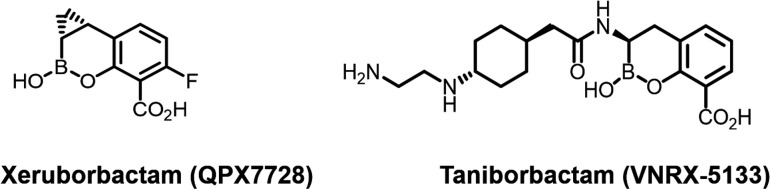
Structures of xeruborbactam and taniborbactam.

Xeruborbactam is another cyclic boronate dual SBL/MBL BLI (6) (Fig. 1). Xeruborbactam has completed Phase 1 studies (NCT04380207, NCT04578873) administered by the IV or oral route (as a prodrug form). In these studies, xeruborbactam was found to be safe and well tolerated at exposures that exceeded non-clinical PK-PD targets (7 - 9). The spectrum of beta-lactamase inhibition by xeruborbactam is the broadest among marketed BLIs and those in clinical development (10). Compared to taniborbactam, xeruborbactam has a broader MBL inhibition spectrum, which includes MBLs of the IMP-type that are not inhibited by taniborbactam (11, 12). The objective of our study was to investigate whether NDM-9 is susceptible to inhibition by xeruborbactam.

Four clinical isolates of *Klebsiella pneumoniae* and one isolate of *Escherichia coli* producing NDM-9 (and other beta-lactamases, Table 1) were included in this study; NDM-1, NDM-4, NDM-5, NDM-6, and NDM-7-producing isolates were added for comparison. Nine out of 13 isolates used in this study were collected by IHMA (International Health Management Associates, Schaumburg, IL)

or JMI (Jones Microbiology Institute, North Liberty, IA) as a part of various worldwide surveillance studies. KP1280 (0106), (KP1297 (0143), EC1100 (0128), and EC1104 (0151) were obtained from the CDC & FDA Antibiotic Resistance Isolate Bank. Isolates were tested for antimicrobial susceptibility using the broth microdilution methodology per Clinical and Laboratory Standards Institute M07 (2018) guidelines (13). The MICs of meropenem and cefepime in combination with xeruborbactam (fixed 4 µg/mL or 8 µg/mL) and taniborbactam (fixed 4 µg/mL) were determined using this panel. Xeruborbactam at both concentrations enhanced *in vitro* potency of meropenem and cefepime against all the NDM-producing strains including those producing NDM-9 (Table 1). Meropenem MIC values were reduced by xeruborbactam (fixed 8 µg/mL) from 16 to >64 µg/mL to ≤0.06 µg/mL for 9 out of 13 tested isolates, including three of five NDM-9 producing strains. Meropenem-xeruborbactam (8 µg/mL) MIC values for the remaining four strains (two with NDM-9- and two with NDM-1) ranged from 1 to 8 µg/mL. These four strains carried various mutations affecting expression or functionality of the major porins OmpF/OmpK35 and/or OmpF/OmpK36 that have previously been demonstrated to restrict meropenem and xeruborbactam entry (14) and have been associated with the increase in meropenem-xeruborbactam MIC (15) (Table 1). No such mutations were present in the strains with meropenem-xeruborbactam (8 µg/mL) MIC values of ≤0.06 µg/mL. Meropenem-xeruborbactam and cefepime-xeruborbactam MIC values were highly correlated: same strains that had low meropenem-xeruborbactam (8 µg/mL) MIC values also had low cefepime-xeruborbactam (8 µg/mL) values (≤0.06 µg/mL, reduced from 32 to >64 for the cefepime alone), including NDM-9-producing strains and vice versa. The only exceptions were the NDM-1 or NDM-5-producing strains of *E. coli*, EC100 and EC1104, respectively, with the low meropenem-xeruborbactam (8 µg/mL) and an increased cefepime-xeruborbactam (8 µg/mL) MIC values. These strains carried four amino acids, YRIN, insertion in PBP3 associated with a decreased susceptibility of cephalosporins and monobactams but not of meropenem (16, 17). Of note, these strains were also resistant to cefepime-taniborbactam (MIC value of 32–64 µg/mL) consistent with recent reports (18, 19). Based on microbiological results, we conclude that xeruborbactam inhibits all the tested NDM variants, including the NDM-9 variant, and that increased MIC values of xeruborbactam combinations are due to the presence of non-beta-lactamase-mediated resistance mechanisms, including porin and/or PBP mutations. As expected, taniborbactam did not enhance activity of either meropenem or cefepime against any of the NDM-9-producing strains but demonstrated enhancement of potency of these antibiotics against isolates producing other NDM variants.

**TABLE 1:** *In vitro* potency (MIC, µg/mL) of meropenem and cefepime in combination with xeruborbactam and taniborbactam against clinical isolates of *Klebsiella pneumoniae* or *Escherichia coli* producing various variants of New Delhi metallo-beta-lactamase^*c*^

Strain	Beta-lactamases	Isolation country/year	OmpK35/OmpF	OmpK36/OmpC	MEM	MEM + XER at 4 µg/mL	MEM + XER at 8 µg/mL	MEM + TAN at 4 µg/mL	FEP	FEP + XER at 4 µg/mL	FEP + XER at 8 µg/mL	FEP + TAN at 4 µg/mL
KP1643	SHV-OSBL; TEM-OSBL; CTX-M-15; **NDM**-1	Mexico, 2017	FL (FN)	FL (FN)	32	≤0.06	≤0.06	0.5	64	≤0.06	≤0.06	0.5
EC1100^*a*^	CMY-6, CTX-M-15, **NDM**-1, OXA-2, TEM-1B-like	USA, 2015	FS at AA#31 (NF)	FL (FN)	>64	0.25	≤0.06	1	>64	16	4	64
KP1297^*b*^	CMY-4, CTX-M-15, DHA-1-like, **NDM**-1, OXA-9, SHV-1-like, TEM-1B-like	USA, 2015	FL, LE (NF)	GD (PFN)	>64	8	2	16	>64	16	4	16
KP1280	**NDM**-1 OXA-1/30 OXA-9 TEM-1 CTX-M-15 SHV-11	USA, 2015	FL, LE (NF)	GD (PFN)	>64	32	4	64	>64	64	8	>64
KP1624	SHV-12; TEM-OSBL; CTX-M-15; **NDM**-4	Vietnam, 2015	FL (FN)	FL (FN)	>64	≤0.06	≤0.06	0.5	32	≤0.06	≤0.06	1
EC1104^*b*^	CMY-42, CTX-M-15, **NDM**-5, OXA-1, SHV-12, TEM-1B-like	USA, 2015	FL (FN)	FL (FN)	64	0.25	≤0.06	2	>64	8	2	32
EC1222	SHV-12; TEM-52; CTX-M-15; CTX-M-27; **NDM**-6	Guatemala, 2016	FL (FN)	FL (FN)	64	≤0.06	≤0.06	0.5	64	≤0.06	≤0.06	0.5
KP1616	SHV-OSBL; TEM-OSBL; CTX-M-15; **NDM**-7	Kuwait, 2015	FL (FN)	FL (FN)	>64	≤0.06	≤0.06	2	>64	0.125	≤0.06	1
KP1690	SHV-ESBL(e); TEM; CTX-M-15; CMY-2-TYPE; **NDM**-9	Guatemala, 2019	FL (FN)	FL (FN)	16	≤0.06	≤0.06	16	32	≤0.06	≤0.06	32
KP1691	SHV-OSBL(b); TEM-OSBL(b); CTX-M-1–240G; **NDM**-9	Ukraine, 2021	FL (FN)	FL (FN)	32	≤0.06	≤0.06	32	32	≤0.06	≤0.06	32
2063549	TEM-OSBL(b); CTX-M-15; **NDM**-9	India, 2019	ND	ND	>16	≤0.06	≤0.06	ND	>32	ND	ND	64
EC1260	CTX-M-65, **NDM**-9, TEM-1	USA, 2015	FL (FN)	FS at aa#113 (NF)	>32	4	1	>32	>32	32	16	>32
KP1658	SHV-OSBL; CTX-M-15; **NDM**-9	Guatemala, 2017	DEL of 670 bp from nt#513 (NF)	GD (PFN)	>64	32	8	>64	>64	16	16	>64

^*a*^ KP1297 and KP1280 have K9I amino acid substitution in *ramR* which results in downregulation of the ompK35 gene and overexpression of the *acrAB* efflux operon.

^*b*^ EC1100 and EC1104 have YRIN duplication in PBP3 (reduced affinity to cephalosporins) and a non-functional repressor of *acrAB* efflux operon, AcrR (the frame-shift from amino acid 27).

^*c*^ NDM variants are shown in bold font; KP, *Klebsiella pneumoniae* isolate; EC, *Escherichia coli* isolate. FL, full length (assumed as functional protein, FN); LE, low expression (assumed as non-functional, NF); GD, a duplication of two amino acids, Gly134Asp135 located within the L3 internal loop resulting in the constriction of the channel (assumed partially functional, PFN); DEL, deletion; FS, frame-shift; aa, amino acid; bp, base-pairs; nt, nucleotide; MEM, meropenem; FEP, cefepime: XER, xeruborbactam; TAN, taniborbactam.

Inhibition of NDM-9 by xeruborbactam was also demonstrated in biochemical experiments. We determined IC_50_ of xeruborbactam (and taniborbactam) inhibition of imipenem hydrolysis in cell lysates prepared from either NDM-1 (KP1297) or NDM-9 (KP1671) producing strains. Bacterial lysates were prepared as previously described (20); imipenem was used at a final concentration of 100 µL. Absorbance profiles at 295 nm (as a result of imipenem cleavage) were recorded every 10 s for 30 min using Tecan plate reader. Initial reaction rates were calculated in OD/min and used to generate dose response curves vs BLI concentration. IC_50_ values of BLI effect on imipenem cleavage were calculated by fitting the resulting curves in “dose-response—inhibition, variable slope (four parameters)” equation using Prizm software.

The IC_50_s of xeruborbactam inhibition of imipenem hydrolysis by NDM-1 (0.77 ± 0.12 µM) or NDM-9 (1.2 ± 0.1 µM) were essentially the same. Taniborbactam inhibited NDM-1-meditaed hydrolysis (IC_50_ = 0.24 ± 0.052 µM) but not NDM-9-mediated hydrolysis (IC_50_ >60 µM).

**Fig 2:**
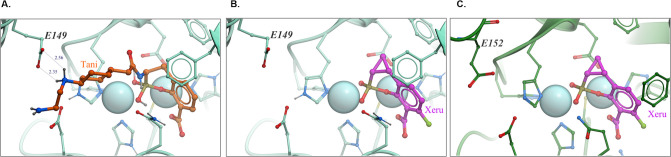
Structural basis of the NDM-9-mediated resistance to taniborbactam and sensitivity to xeruborbactam: x-ray structures of VIM-2 with taniborbactam and xeruborbactam and NDM-1 with xeruborbactam. (A)VIM-2 in complex with taniborbactam (PDB reference 6SP7); (B) VIM-2 in complex with xeruborbactam (PDB reference 6V1P); (C) NDM-1 in complex with xeruborbactam (PDB reference 6P1M). E149 in VIM-2 (corresponds to E152 in NDM-1) forms a salt bridge with the positively charged amine of the taniborbactam side chain (panel A). In NDM-9, the negatively charged glutamic acid (E152) is changed to a positively charged lysine (E152K). The E to K substitution observed in NDM-9 or the VIM mutant [generated by Le Terrier et al. (4)] turns the previously favorable interaction between taniborbactam and the enzyme at this position into repulsion, which explains resistance to taniborbactam inhibition. In contrast, xeruborbactam molecule doesn’t rely on this interaction at E149 in VIM-2 (panel B) or E152 in NDM-1 (panel C) and is predicted to not be affected by mutations in these positions.

Available crystallography data (6, 12) provide a clear explanation for why the NDM-9 variant that differs from NDM-1 by a single E152K substitution is resistant to the inhibition by taniborbactam but remains susceptible to inhibition by xeruborbactam. In NDM-1, the negatively charged glutamic acid (E152) (corresponds to E149 in VIM-2) forms a salt bridge with the positively charged amine of the taniborbactam sidechain (Fig. 2). In NDM-9, E152 is substituted by a positively charged lysine, E152L. This E to K substitution turns the previously favorable interaction between taniborbactam and the enzyme at this position into repulsion, which explains resistance to taniborbactam inhibition. In contrast, the xeruborbactam molecule doesn’t rely on this interaction and is predicted to not be affected by mutations in these positions.

In conclusion, we have shown that xeruborbactam retains high potency against NDM-9 as well as against other NDM variants (e.g*.*, NDM-4 and NDM-6) that also evolved to withstand Zn (II) deprivation (1). This finding highlights the differences between boronic-acid-based dual spectrum BLIs and underscores the importance of further development of xeruborbactam.
